# Functional connectivity of the cortico-subcortical sensorimotor loop is modulated by the severity of nigrostriatal dopaminergic denervation in Parkinson’s Disease

**DOI:** 10.1038/s41531-022-00385-w

**Published:** 2022-09-28

**Authors:** Mario Quarantelli, Andrea Quattrone, Alessia Sarica, Francesco Cicone, Giuseppe Lucio Cascini, Aldo Quattrone

**Affiliations:** 1 Institute of Biostructure and Bioimaging, National Research Council, Naples, Italy; 2 Department of Medical and Surgical Sciences, Institute of Neurology, “Magna Graecia” University, Catanzaro, Italy; 3 Neuroscience Research Center, “Magna Graecia” University, Catanzaro, Italy; 4 Nuclear Medicine Unit, “Mater Domini” University Hospital, Catanzaro, Italy; 5 Department of Experimental and Clinical Medicine, “Magna Graecia” University, Catanzaro, Italy; 6 Neuroimaging Research Unit, Institute of Molecular Bioimaging and Physiology (IBFM), National Research Council (CNR), Catanzaro, Italy

**Keywords:** Parkinson's disease, Magnetic resonance imaging, Molecular imaging, Motor control

## Abstract

To assess if the severity of nigrostriatal innervation loss affects the functional connectivity (FC) of the sensorimotor cortico-striato-thalamic-cortical loop (CSTCL) in Parkinson’s Disease (PD), Resting-State functional MRI and ^18^F-DOPA PET data, simultaneously acquired on a hybrid PET/MRI scanner, were retrospectively analyzed in 39 PD and 16 essential tremor patients. Correlations between posterior Putamen DOPA Uptake (pPDU) and the FC of the main CSTCL hubs were assessed separately in the two groups, analyzing the differences between the two groups by a group-by-pPDU interaction analysis of the resulting clusters’ FC. Unlike in essential tremor, in PD patients pPDU correlated inversely with the FC of the thalamus with the sensorimotor cortices, and of the postcentral gyrus with the dorsal cerebellum, and directly with the FC of pre- and post-central gyri with both the superior and middle temporal gyri and the paracentral lobule, and of the caudate with the superior parietal cortex. The interaction analysis confirmed the significance of the difference between the two groups in these correlations. In PD patients, the post-central cortex FC, in the clusters correlating directly with pPDU, negatively correlated with both UPDRS motor examination score and Hoehn and Yahr stage, independent of the pPDU, suggesting that these FC changes contribute to motor impairment. In PD, nigrostriatal innervation loss correlates with a decrease in the FC within the sensorimotor network and between the sensorimotor network and the superior temporal cortices, possibly contributing to motor impairment, and with a strengthening of the thalamo-cortical FC, that may represent ineffective compensatory phenomena.

## Introduction

Several studies have demonstrated the presence in Parkinson’s Disease (PD) patients of alterations of the Functional Connectivity (FC) of the hubs of the cortico-striato-thalamo-cortical loop (CSTCL)^1–4^. However, the reported patterns of FC alterations are inconsistent, and in particular do not provide evidence for systematic alterations in the FC of the hubs of the sensorimotor network^1,5^.

Among the main potential confounders in these studies, there is the variability of the residual nigrostriatal dopaminergic tone, which is influenced by both the degree of nigrostriatal neuronal loss and by the ongoing treatment status^2^.

Indeed, the acute direct effect of dopamine on striatal and cortical FC was demonstrated by resting-state functional MRI (RS-fMRI) studies that compared the ON and OFF conditions. The administration of L-DOPA to PD patients was shown to change functional connectivity (FC) both within the sensorimotor network^6–8^, and between motor areas and non-motor networks^7,9^. Although with somewhat different results across studies, in general, the L-DOPA treatment has shown to rebalance the brain connectivity acting on multiple hubs, with a wide range of influences in the same direction of clinical effectiveness^2,10,11^.

Despite this body of evidence demonstrating an impact of dopamine on RS-FC in PD, very limited data are available on the relationship between the FC modifications of the CSTCL and the degree of the reduction in the dopaminergic tone that characterizes PD.

Dopaminergic striatal innervation can be studied in vivo by Positron Emission Tomography (PET) or Single-Photo Emission Tomography (SPECT) using either labelled L-DOPA as tracer of dopamine synthesis, or labelled dopamine transporter ligands as presynaptic markers of dopaminergic terminals, while functional connectivity can be studied using RS-fMRI. The integration of the information provided by these modalities allows assessing in-vivo the relationship between nigrostriatal innervation integrity and FC, by exploiting the different degrees of loss of nigrostriatal neurons that occur across different patients. However, this approach has been so far limited to the study of the relationship between the dopaminergic innervation of the putamen and the FC of the same structure^12–14^, or of the posterior hub of the default-mode network^12^.

The recently introduced hybrid PET/MRI scanners are particularly suited to study dopaminergic striatal innervation and FC simultaneously. In particular, the simultaneous dynamic acquisition of T2*-weighted Echo-Planar Imaging (EPI) volumes and PET data, beside allowing to improve the quality of the PET data by their framewise prospective motion correction^15^, provides a set of RS-fMRI data suitable for FC analysis.

The aim of our study was to assess if, in PD patients in practical OFF condition, the reduction in the nigrostriatal innervation, as probed by 3,4‑dihydroxy‑6‑[^18^F]‑fluoro-l‑phenylalanine (^18^F-DOPA) uptake in the posterior putamen, affects the FC of the hubs of the sensorimotor CSTCL, as simultaneously probed by resting-state functional MRI.

To this end, RS-fMRI and ^18^F-DOPA PET data, simultaneously acquired on a hybrid PET/MRI scanner, were retrospectively analyzed in a group of PD patients and in a control group of patients with essential tremor (ET), a disease in which nigrostriatal innervation is preserved.

From PET data, ^18^F-DOPA uptake in the posterior putamen (posterior Putamen DOPA Uptake - pPDU) was measured. Concurrently, a seed-based analysis of the RS-fMRI data acquired simultaneously to PET was carried out to assess brain-wise the FC of each of the main hubs of the sensorimotor CSTCL.

Correlation between the FC maps of the CSTCL seeds and the pPDU was assessed voxelwise over the whole brain separately for the two groups.

Where clusters of significant correlations emerged in any of the two groups, a group-by-pPDU interaction analysis of the corresponding mean FC values was carried out, to assess if the correlation was significantly different between the PD and ET groups.

Finally, the correlation of the FC in these regions with the degree of motor impairment, as measured by the pars III (motor examination) of the Unified Parkinson’s Disease Rating Scale^16^ (UPDRS-ME) and by the Hoehn and Yahr stage^17^ (H&Y), was assessed by linear regression analysis.

## Results

### Demographics and clinical data of patient groups

PET/MRI studies from 39 PD and 16 ET patients fulfilled inclusion and exclusion criteria. Clinical data of the two groups are reported in Table 1.

**Table 1:** Composition of PD and ET patient groups.

	PD	ET
*n*	39	16
M/F	28/11	9/7
Age (y)		
Mean	64.8	72.3
SD	7.4	8.8
(range)	(49–79)	(50–85)
Education (y)		
Mean	10.7	7.9
SD	4.0	2.9
(range)	(3–17)	(4–13)
MMSE		
Mean	24.7	23.9
SD	4.3	3.8
(range)	(16–30)	(15–30)
Disease Duration (y)		
Mean	5.3	n/a
SD	3.9	
(range)	(0–18)	
^a^UPDRS-I score		
Mean	8.6	n/a
SD	4.2	
(range)	(1–18)	
^a^UPDRS-II score		
Mean	7.5	n/a
SD	6.0	
(range)	(1–26)	
^a^UPDRS-III (ME) score		
Mean	20.4	n/a
SD	12.6	
(range)	(4–54)	
^a^UPDRS-IV score		
Mean	1.1	n/a
SD	2.8	
(range)	(0–12)	
^a^Hoehn and Yahr stage		
Mean	1.6	n/a
SD	0.6	
(range)	(1–3)	

All clinical scores were measured within one week from the PET study in OFF condition.

*MMSE* Mini-Mental Status Examination Score.

^a^UPDRS score and H&Y stage were available in 35 PD patients.

PD patients were significantly younger (64.8 vs. 72.3, *p* = 0.004 at t-test) and had more years of education (10.7 vs. 7.9, *p* = 0.02 at Mann-Whitney test) compared to the ET patients.

No significant differences were present between the two groups in terms of sex (at Chi-squared), and Mini-Mental State Examination (MMSE^18^) score (at Mann-Whitney test).

pPDU was significantly lower in PD compared to the ET patients (1.8 ± 0.3 vs. 3.1 ± 0.6, *p* < 10^−13^), and correlated inversely with the Hoehn and Yahr stage (H&Y, *p* = 0.018), with only a trend for inverse correlation with the UPDRS- ME (*p* = 0.098).

### Correlations between FC and pPDU

No significant correlation emerged in ET patients between pPDU and FC of any of the CSTCL seeds.

**Fig. 1:**
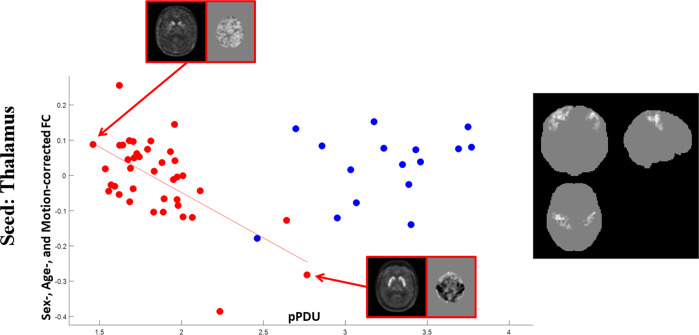
Correlations of thalamic FC with pPDU. Scatterplots for PD (red) and ET (blue) patients of the mean FC of the clusters (highlighted in the inserts on the right, the most affected side is at reader’s left) showing a significant inverse correlation of the thalamic FC (*y* axis) with pPDU (*x* axis) in PD patients. For the purposes of the graph, all clusters’ voxels were averaged as all the clusters from this contrast provided substantially overlapping patterns of correlation. FC of the thalamus on the MAS with bilateral rolandic cortices shows an inverse correlation with pPDU in PD patients, with no significant correlations in ET patients in the same regions. For demonstrative purposes, the FC maps at the level of the bilateral sensorimotor cortex of the PD patients at the extreme of the pPDU range are shown. A clear negative thalamic FC in the sensorimotor strips in the PD patient with the highest pPDU can be appreciated.

**Fig. 2:**
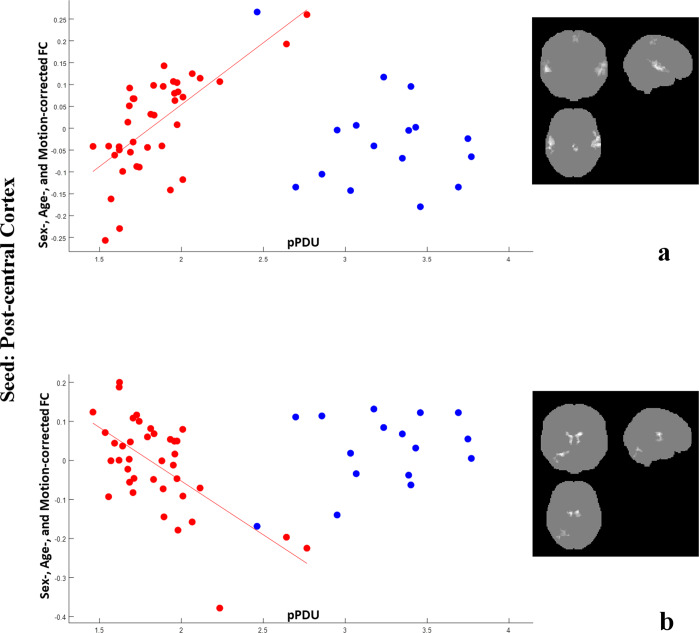
Correlations of post-central cortex FC with pPDU. Scatterplots for PD (red) and ET (blue) patients of the mean FC of the clusters (highlighted in the inserts on the right, the most affected side is at reader’s left) showing a significant correlation of the post-central cortex FC (y axis) with pPDU (*x* axis) in PD patients. For the purposes of the graph, for each contrast all clusters were averaged as they provided substantially overlapping patterns of correlation. The FC of the post-central cortex on the MAS with the superior/mid temporal gyri and the paracentral lobule shows a significant direct correlation with pPDU in PD patients, with no significant correlation in ET patients in the same regions (**a**). On the contrary, The FC between the post-central cortex homolateral to the MAS and both thalami and homolateral lobule 6 and crus 1 of the cerebellum decreases with increasing pPDU in PD patients, also in this case without a significant correlation in ET patients (**b**).

**Fig. 3:**
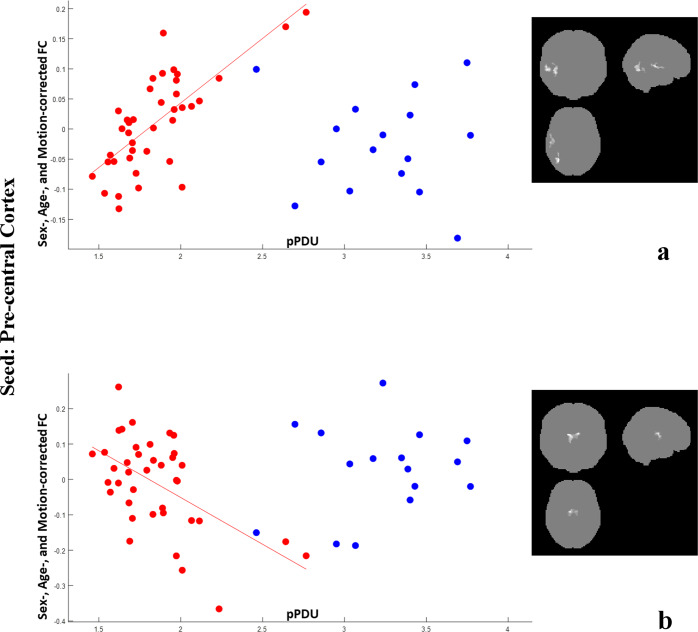
Correlations of pre-central cortex FC with pPDU. Scatterplots for PD (red) and ET (blue) patients of the mean FC of the clusters (highlighted in the inserts on the right, the most affected side is at reader’s left) showing a significant correlation of the pre-central cortex FC (*y* axis) with pPDU (*x* axis) in PD patients. For the purposes of the graph, for each contrast all clusters were averaged as they provided substantially overlapping patterns of correlation. The FC of the pre-central cortex on the MAS with the homolateral superior/mid temporal gyri and inferior occipital cortex shows a significant direct correlation with pPDU in PD patients, with no significant correlation in ET patients in the same regions (**a**). On the contrary, The FC of the pre-central cortex on the MAS with the thalami decreased with increasing pPDU in PD patients (**b**).

**Fig. 4:**
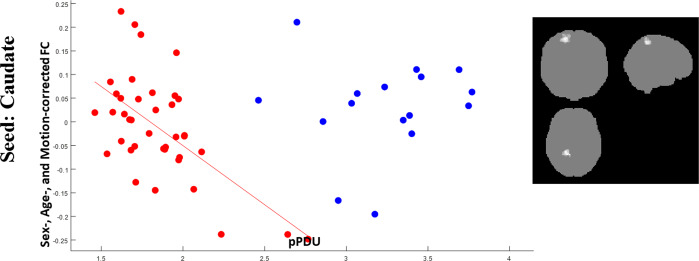
Correlations of post-central cortex FC with pPDU. Scatterplots for PD (red) and ET (blue) patients of the mean FC of the cluster (highlighted in the inserts on the right, the most affected side is at reader’s left) showing a significant correlation of the caudate FC (*y* axis) with pPDU (*x* axis) in PD patients. FC of the caudate on the MAS with the homolateral superior parietal cortex shows an inverse correlation with pPDU in PD patients, with no significant correlation in ET patients in the same regions.

Several clusters of significant correlation between pPDU and the FC of the main hubs of the sensorimotor CSTCL were present in PD patients, and are reported in Table 2, and shown along with the scatterplots of the corresponding mean FC values in Figs. 1–4. Note that for the purposes of these figures, for each contrast all clusters’ voxels are averaged as all the clusters from each contrast have substantially overlapping mean FC values.

**Table 2:** Clusters showing a significant correlation of FC with pPDU in PD patients.

Seed	Correlation	*p*	Size (cm3)	T	MNI Coordinates[XYZ]	Side	AAL	Interaction (p value)	Correlation with UPDRS-ME (*p* value)	Correlation with H&Y stage (*p* value)
Thalamus	Inverse	<5·10^−10^	13.2	5.78	[26;−36;54]	Homolateral	Postcentral	<5·10^−5^	n.s.	n.s.
				5.19	[48;−20;44]	Homolateral	Precentral			
		<5·10^−9^	5.6	5.12	[−48;−8;36]	Contralateral	Precentral			
				5.08	[−44;−30;62]	Contralateral	Postcentral			
Post-Central Gyrus	Inverse	0.0004	2	5.09	[−10;−8;12]	Contralateral	Thalamus	<5·10^−5^	n.s.	n.s.
		0.0013	1.7	4.86	[8;−12;0]	Homolateral	Thalamus			
		0.0012	1.7	4.21	[26;−60;−32]	Homolateral	Cerebellum 6			
				4.1	[30;−70;−30]	Homolateral	Cerebellum Crus 1			
	Direct	<5·10^−7^	4.5	7.7	[56;−14;−6]	Homolateral	Temporal Sup	<5·10^−5^	0.0073	0.0017
		<5·10^−9^	6	6.21	[−68;−24;8]	Contralateral	Temporal Sup			
				5.53	[−50;−24;−2]	Contralateral	Temporal Mid			
		0.0048	1.4	4.64	[−6;−36;60]	Contralateral	Paracentral lobule			
Pre-Central Gyrus	Inverse	0.0003	2.1	4.59	[4;−14;8]	Homolateral	Thalamus	0.0018	n.s.	n.s.
	Direct	0.0008	1.8	5.09	[40;−72;−8]	Homolateral	Occipital Inf	0.0002	n.s.	n.s.
				4.23	[38;−54;−10]	Homolateral	Fusiform			
				4.14	[46;−62;8]	Homolateral	Temporal Mid			
		0.0019	1.6	4.97	[54;−16;−6]	Homolateral	Temporal Sup			
Caudate	Inverse	<5·10^−6^	3.3	5.42	[18;−46;62]	Homolateral	Parietal Sup	0.0016	n.s.	n.s.
				4.53	[10;−52;70]	Homolateral	Precuneus			

For each cluster the *p* value (FWE-corrected at cluster level) is reported, along with the corresponding volume, T value, and coordinates in the MNI space of the cluster’s local maxima.

Anatomical labeling are reported according to ref. ^50^.

For each contrast, significance of the difference between the slopes in the PD and ET patient groups at the interaction analysis is also reported for the FC averaged over of the corresponding cluster set, along with significance of the correlation with the clinical scores.

No significant correlations emerged between FC and pPDU in ET patients.

In particular, FC between the thalami and the bilateral rolandic cortices, as well as between the post-central cortex and the homolateral dorsal cerebellum (mainly lobule 6, part of the sensorimotor network^19^) showed an inverse correlation with pPDU in PD patients (Figs. 1, 2b, and 3b).

An opposite behavior was found for the FC of the pre- and post-central gyri with the middle and superior temporal gyri, and of the post-central cortex with the homolateral inferior occipital cortex, including the fusiform gyrus, which increases significantly with pPDU in PD patients (Figs. 2a and 3a).

Finally, FC of the caudate with a cluster located in the homolateral superior parietal cortex/precuneus increased with increasing loss of nigrostriatal innervation in PD patients (Fig. 4).

No other significant correlations emerged for the other seeds.

Thus, in PD patients a total of six contrasts resulted in clusters of significant correlation between FC and pPDU for four seeds (pre- and post-central gyri showing both clusters of direct and inverse correlation). Accordingly, for the subsequent interaction and clinical correlation analyses, which were carried out for each contrast on the FC averaged over all the corresponding clusters, *p* ≤ 0.0083 (i.e. 0.05 Bonferroni-corrected for six contrasts) was considered significant.

The interaction analysis confirmed in all cases a significant difference between the PD and ET groups in the correlation of FC with pPDU (“Interaction” column in Table 2).

### Correlations between FC and clinical data

**Fig. 5:**
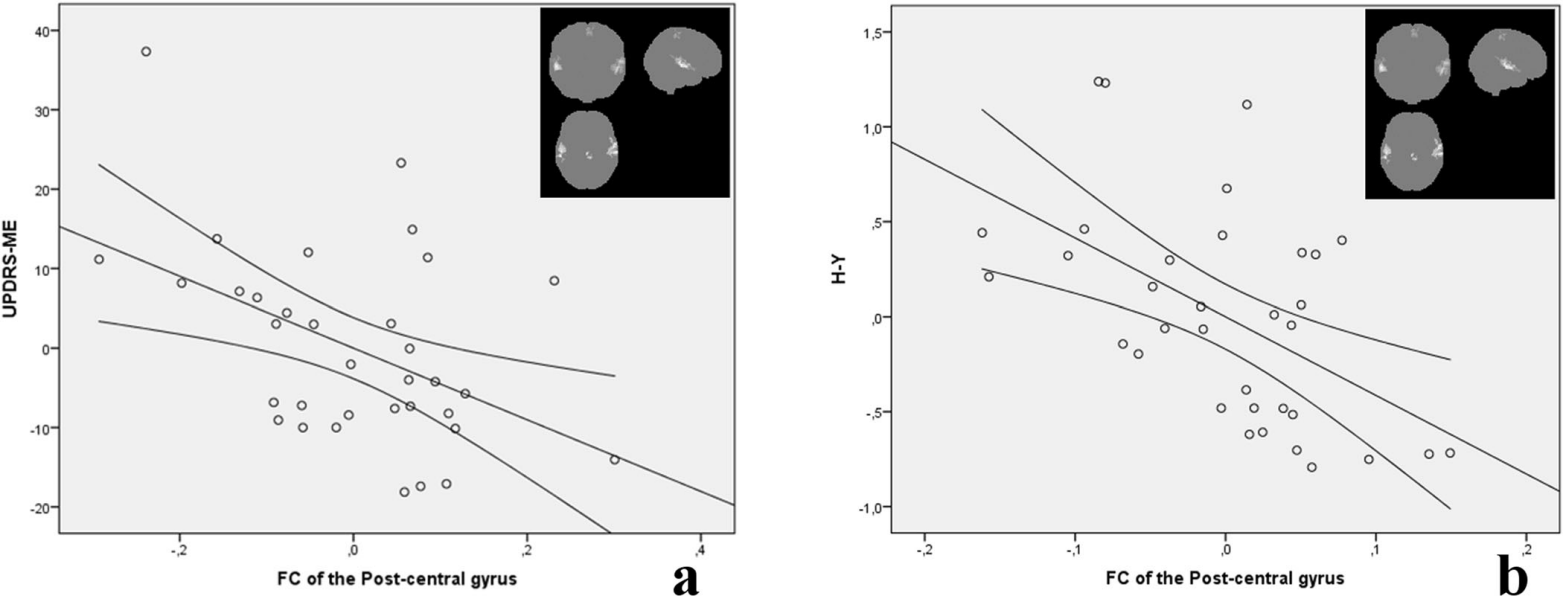
Correlations of post-central cortex FC with clinical scores. Scatterplots of the Unified Parkinson’s Disease Rating Scale Motor Examination score (UPDRS-ME, **a**, *p* = 0.007) and Hoehn and Yahr stage (H&Y, **b**, *p* = 0.002) against the mean FC of the regions where the post-central cortex FC correlates with pPDU (highlighted in the inserts on the right, the most affected side is at reader’s left). For each graph, residuals from the linear regression analysis, following correction for age sex and motion parameters, are plotted. Regression line along with the 95% confidence intervals is superimposed.

When testing the correlation of the FC with the clinical scores in PD patients, the mean FC of the clusters where the FC of the post-central cortex showed a significant direct correlation with pPDU in PD patients, correlated inversely with the UPDRS-ME (Fig. 5a, *p* = 0.007) and H&Y (Fig. 5b, *p* = 0.002).

In both cases, the correlation with the clinical scores remained significant also when including pPDU in the model (*p* = 0.02 and 0.002 for UPDRS-ME and H&Y, respectively).

## Discussion

We have assessed the correlation between the residual dopamine synthesis capacity in the posterior putamen, and the FC of the main hubs of the sensorimotor cortico-striatal-thalamo-cortical loop in PD patients, as compared to ET patients.

Our results demonstrate an inverse correlation in PD patients in the OFF condition between the pPDU and the FC of the thalamus with the sensorimotor cortices, and of the caudate with the superior parietal cortex. Simultaneously, a significant direct correlation with the nigrostriatal innervation of the FC between different nodes of the sensorimotor network, as well as between the sensorimotor cortices and the superior temporal cortices could be detected. Contrary to PD, no significant correlations emerged in the ET patients, and the interaction analysis confirmed the specificity to PD of this pattern of correlations and anticorrelations.

Previous studies of the relationship between nigrostriatal dopaminergic innervation and FC have focused on striatal FC. In particular, the correlation of the nigrostriatal innervation, sampled by the striatal TRODAT-1 binding potential^14^, ^11^C-Altropane PET^13^, ^11^C-PE2I^20^, or with ^18^F-FP-CIT PET^12^, with the FC of the striatum has been previously studied in patients with nigrostriatal dopamine loss. Results showed in de-novo, drug-naive PD patients a direct correlation between the posterior putamen dopaminergic innervation and its FC with the dorsolateral frontal cortex contralateral to the MAS, and with the dorsal cerebellum bilaterally^12^. On the contrary, a study carried out in more advanced PD patients^14^ with a disease duration comparable to our PD group, failed to detect any correlation between the striatal TRODAT uptake and the FC of the posterior putamen, in line with our data.

Alternatively, the effect of dopamine depletion on FC between putamen and sensorimotor cortex has been explored in a similar setting, using an ROI-based approach to the analysis of the putaminal FC^13^. In that case, the analysis was limited to a set of pre-defined ROIs, and carried out in a heterogeneous group of PD patients with a longer disease duration, including patients with dementia with Lewy bodies. Using this approach, a trend for an inverse correlation between the residual dopaminergic innervation and the FC between the putamen and the postcentral gyrus was found.

Accordingly, longitudinal studies are needed to clarify if this specific correlation with putaminal FC is indeed related to disease duration.

Interestingly, a recent longitudinal study in a small group of PD patients^20^ demonstrated highly significant correlations between changes over time in putaminal dopamine transporter density and the changes in FC of the posterior putamen with midbrain, thalamus, supplementary motor area and sensorimotor cortex. In the same study weaker and spatially limited correlations were found at baseline between dopamine transporter density in the posterior putamen and its FC with adjacent regions and the midbrain, suggesting that individual variability plays a major role in our ability to detect these associations.

In the present work, we have expanded this approach, specifically targeting the correlations between the pPDU and the FC of the main hubs of the sensorimotor CSTCL (pre- and post-central gyri, posterior putamen, globus pallidus, and thalamus). In addition, we have included a control group without deficits of the nigrostriatal dopaminergic innervation, to assess the specificity to PD of any correlation that could emerge.

Our results confirm a significant effect of DOPA on the FC of the sensorimotor network in PD, in line with the evidences for a role of nigrostriatal innervation in modulating cortical FC derived from neuroimaging studies of pharmacological intervention in humans. In particular, previous works analyzing FC changes associated to the transition from the OFF to the ON state in PD have highlighted the capacity of DOPA to modulate the functional connectivity of the sensorimotor network^7,8,21^. In those studies, the intake of L-DOPA increased functional connectivity within the sensorimotor network, as well as between motor areas and middle temporal gyri^8^, dorsal-attention and default-mode networks^7^. These results are in agreement with the pattern emerging from our study of increasing FC within the sensorimotor cortex (indicated by the paracentral lobule cluster of direct correlation between pPDU and FC of the postcentral cortices) and between the sensorimotor cortex and the superior and middle temporal gyri in PD patients with higher pPDU values.

Interestingly, a more extensive integration of the superior temporal cortex in the sensorimotor network has been shown to play a role in the adaptive processes during ageing, correlating with grip force and finger tapping frequency^22^.

On the other hand, also in line with current data, previous results of pharmacological challenges have also provided evidences for an inverse effect of DOPA administration on thalamo-cortical FC. In particular, when analyzing FC of motor cortex subregions, an L-DOPA-induced reduction in FC between upper limb region and the medial dorsal thalamus emerged^8^, in line with the inverse correlation we found between pPDU and the FC of the thalamus with the rolandic cortices.

The higher FC between thalamus and sensorimotor cortex in patients with most severe loss of dopaminergic neurons, is also in line with previous studies showing a reduced thalamocortical connectivity in early PD^23^ and increased FC of the motor thalamus in more advanced PD patients off therapy^24^.

It should be noted however that the effects on the motor circuits’ FC of the chronic dopamine depletion that occurs in PD patients have been derived indirectly in the studies discussed above. Estimates of the effect of dopaminergic depletion on FC are in those cases inferred from differences detected either between PD patients and controls, or between studies of PD patients in the OFF and ON state. These two approaches however suffer from several limitations that can explain some of the differences between our results and those of the studies discussed above.

In particular, when comparing PD patients and controls, FC is averaged across patients with different dopaminergic neuronal loss. As a consequence, both false positives (for regions with FC changes not directly related to dopamine depletion), and false negatives (for regions where dopamine-modulated FC remains within its normal limits, a pattern present in most of our results) may occur. On the other hand, FC differences between PD patients in the ON and OFF condition, probe the acute and subacute effects of dopamine on depleted systems, rather than the direct effects of the chronic depletion, ignoring the adaptive mechanisms occurring in the brain.

The approach implemented in the present work on the contrary allows to assess the direct consequences of chronic dopaminergic neuronal degeneration, typical of PD, on the FC.

Although PD is a neurodegenerative disease, only part of the symptoms is directly linked to dopaminergic neuronal loss, and the role of FC alterations in determining the remaining symptom burden is increasingly evident^25,26^. From this standpoint, the clearer understanding of how the loss of dopaminergic innervation influences the balance of cortico-subcortical loops, provided by the current approach, adds to the current knowledge of FC alterations in PD, and has potential to promote the development of new disease monitoring processes and therapeutic intervention strategies.

Indeed, current results are in line with a recent meta-analysis of seed-based resting-state fMRI studies of the CSTCL in PD^27^, which reported as main alteration the hyperconnectivity of the post-central cortex. In that work, the lack of correlation of post-central FC with disease duration or severity of symptoms confirmed a more direct relationship with the core neuropathological bases of the disease, in line with the general view that sensory deficits are likely to be a consequence of the dopaminergic denervation of the striatum^28^. Accordingly, it was suggested that the post-central gyrus may represent a potential alternative target for repetitive transcranial magnetic stimulation in PD. Current results, while confirming the direct correlation of FC alterations in the post-central gyrus with nigrostriatal dopamine depletion, disclose specific correlations with disease severity of the FC of the postcentral cortex with the superior temporal gyri. These additional regions could provide new targets for the neuromodulatory therapies, or their FC could be used to monitor directly the efficacy of the TMS on post-central gyri.

In addition, the relationship between dopaminergic depletion and thalamocortical hyperconnectivity is in line with recent data indicating that deep brain stimulation, targeting either the globus pallidus pars interna or the subthalamic nucleus, decreases the FC of the thalamus with the somatosensory-motor cortices^29^. Indeed, hyperactivity of the internal pallidus and subthalamic nucleus is a well-known net effect of the loss of inhibitory nigrostriatal projections in PD. In this context, the present data offer an interpretative frame for those results, suggesting that the suppression by deep brain stimulation of the abnormal hyperactivity of the subthalamic nucleus and the globus pallidum pars interna reverts the effects of dopamine loss, bringing the patients to a thalamocortical FC equilibrium closer to the one that is present in milder stages of the disease.

This interpretation should however be considered cautiously, as other studies of the effects on FC of the deep brain stimulation^30,31^ have suggested an opposite pattern, so that further work is needed to assess the actual pathophysiological meaning of these changes.

Also the higher FC between caudate and precuneus in patients with most severe loss of dopaminergic neurons is in line with previous studies showing a reduced FC along this path in earlier stages^32,33^, with normal FC values in more advanced stages of the disease^33^. It is however not surprising that no clinical correlation was present for the FC of this region, as connectivity between the caudate and the DMN has been associated to cognitive control on impulsive behavior and motivational and automatic processes^23^, that were not tested for the purposes of the present study.

Finally, in considering current results, some limitations should be taken into account.

We did not include normal subjects, to avoid unnecessary exposure to ionizing radiations. It should be however noted that this choice is unlikely to have affected our results, as ET is characterized by normal dopaminergic striatal innervation, as demonstrated by a number of PET and SPECT studies^34^. However, further studies in healthy controls are warranted to properly assess that no significant correlation between pPDU and FC is present under normal conditions of dopaminergic striatal innervation.

Similarly, the retrospective nature of the work did not allow to properly match for age and education of the two groups. To fully exclude the effect of these nuisance covariates on our results, we carried out the same analysis presented here also on a subset of the patients, including 31 PD and 12 ET patients, matched for age (67.4 ± 6.1 vs. 69.0 ± 7.5 years, *p* = n.s.) and education (10.4 ± 4.2 vs. 8.2 ± 3.0 years, *p* = n.s.), which provided substantially identical results as the full dataset (data not shown).

In addition, the potential effects of different pharmacological treatments that were obviously present in the two groups were minimized by a pharmacological washout, according to current relevant guidelines for ^18^F-DOPA PET scans. However, prospective studies in drug-naïve patients are needed to rule out any residual effect of this variable on both pPDU and FC.

Furtnermore, we have used a single thalamic ROI, as the limited number of subjects precluded the possibility to perform at this stage a meaningful analysis of specific thalamic nuclei, which are characterized by different FC patterns^35^. Future studies are warranted to assess if the use of more specific ROIs targeting thalamic nuclei can improve the characterization of the phenomena depicted by our results.

For the purposes of the present work, to maximize the possibility to detect significant correlations, we chose a-priori to focus on the dopaminergic innervation of the posterior putamen (the most consistent and conspicuous alteration in ^18^F-DOPA PET studies of PD, considered responsible for the motor symptoms of the disease), and on the CSTCL motor loop.

However, loss of dopaminergic innervation is detectable by ^18^F-DOPA PET scans in several other structures in PD, including (but not limited to) anterior putamen, caudate and the cortical terminals of the mesocortical dopaminergic system. The use also of these other structures to assess nigrostriatal degeneration by measuring their ^18^F-DOPA uptake would however have proportionally increased the number of analyses, reducing the statistical power of the results due to the further corrections for multiple comparisons. Accordingly, future studies may expand the current work to the remaining dopaminergic nigrostriatal targets. These studies should enroll sufficiently large patient samples to overcome the smaller reductions in ^18^F-DOPA uptake encountered in these other structures, and should include assessment of correlations with non-motor scores (e.g. probing executive and speech dysfunction, visual spatial ability and specific memory impairment), which were not available in our clinical sample.

Also, some caveats specifically related to the use ^18^F-DOPA PET to assess nigrostriatal degeneration must be considered.

Upregulation of aromatic L-amino acid decarboxylase activity (the main responsible for neuronal ^18^F-DOPA uptake) is present in PD. This is a compensatory response to dopaminergic neuronal death that results in a relative underestimation of nigrostriatal degeneration when using ^18^F-DOPA^36^. In addition, other non-dopaminergic neurons are affected in PD that show some degree of ^18^F-DOPA uptake^37^, so that both sensitivity and specificity issues must be considered when using this tracer.

To overcome these limitations, studies similar to the present one, but carried out with ligands for the dopamine transporter, a specific marker of dopaminergic neurons, not hampered by upregulation, are warranted (although some degree of downregulation has been in turn also suggested for these tracers^38^). Also, the effects on FC of relevant structures of the degeneration of non-dopaminergic systems, known to be altered in PD (e.g. serotonergic, cholinergic and noradrenergic systems)^38^, could be specifically studied using the current approach applied to data from PET tracers specific for these neurotransmitters.

Finally, application of the same approach to the FC metrics derived from other advanced fMRI techniques (probing the functional connectome^39^) and from structural connectivity studies (e.g. tractographic diffusion tensor reconstructions directly probing structural integrity of the nigrostriatal tracts^40^), should also be considered, to provide complementary information needed to validate and expand current results.

In summary, our results demonstrate that the FC pattern of the CSTCL is influenced by nigrostriatal innervation in PD. In particular, decreasing pPDU values in PD patients directly correlate with the connectivity of the sensorimotor cortex with the paracentral lobule and superior temporal regions, potentially contributing to the motor function loss typical of the disease, as suggested by correlation with clinical score. A consensual inverse correlation between the pPDU and the FC of the thalamus with the sensorimotor cortices is also present in PD, that may represent ineffective compensatory phenomena.

These findings, which may explain the heterogeneity of previous results of FC studies in PD, suggest the need, when assessing FC alterations in PD, to stratify patients according to the degree of nigrostriatal denervation.

Future longitudinal studies are needed to clarify the role of these modifications in the clinical progression of the disease.

## Methods

### Patients

Clinical records of patients who had undergone an ^18^F-DOPA study within the routine clinical activity on the hybrid PET/MRI scanner at the University “Magna Graecia” of Catanzaro, Italy, from 01-Jul-2017 to 30-Nov-2021 were retrospectively analyzed.

Inclusion criteria were a final diagnosis of PD^41^ or ET^42^ and the availability of a ^18^F-DOPA PET/MRI study of adequate quality, without significant artifacts related to patient motion and/or major magnetic field inhomogeneities (e.g. related to dental implants).

Exclusion criteria for all the subjects were a current or past history of psychiatric or neurological disorders other than PD or ET, or of major head trauma, substance abuse, severe heart, liver, and kidney dysfunction, malignant tumors, major endocrine and metabolic diseases, and treatment with medications active on the CNS other than those for PD or ET.

In addition, subjects were excluded from the analysis if any abnormality was present on the MRI scan, apart from mild age-related leukoaraiosis (i.e. Fazekas score >1^43^).

UPDRS scores, H&Y stage, and MMSE scores, measured in OFF condition, were also recorded if available and collected within 1 week from the PET scan.

The study was conducted in accordance with the ethical standards of the 1964 Declaration of Helsinki and later amendments. Written informed consent was obtained from all patients.

The present study was approved by the local Institutional Review Board of the University “Magna Graecia” of Catanzaro, Italy (Authorization number n. 358, 22 October 2020).

### PET/MRI acquisition

PET and RS-fMRI studies were simultaneously acquired using a hybrid PET/MRI scanner (Biograph mMR, Siemens Healthcare, Erlangen, Germany)^44^, which includes a multi-ring LSO detector block embedded into a 3 Tesla MRI scanner.

PET nominal axial and transverse resolution at 1 cm from the isocenter is 4.4 mm and 4.1 mm FWHM, respectively.

All ^18^F-DOPA PET studies were carried out in compliance with the European Association of Nuclear Medicine guidelines^45^.

Scans were carried out following at least 4 h fasting and discontinuation of any anti-Parkinsonian drug (including L-DOPA, dopamine agonists, monoamine oxidase B inhibitors, N-methyl-D-aspartate receptor blockers, and amantadine) by at least 12 h.

Medications to relieve ET symptoms, which were assumed by seven ET patients (including benzodiazepines, pramipexole, and/or propranolol) were discontinued for at least 48 h before the 18F-DOPA PET scan.

200 mg of carbidopa were administered 1 h prior to ^18^F-DOPA injection, to maximize the availability of DOPA to the brain and reduce the absorbed dose to bladder and kidneys^46^.

Simultaneous brain PET and RS-fMRI data acquisition started 86.1 ± 21.8 min following the i.v. injection of 3 MBq/kg of ^18^F-DOPA (81.4 ± 19.4 min in ET, 86.1 ± 22.9 min in PD patients, difference not significant at t-test).

During the scan, subjects were instructed to relax and keep their eyes closed, without falling asleep. For all studies, PET data were acquired in sinogram mode for 20 min, and data were reconstructed using an ordered subset-expectation maximization algorithm (21 subsets, 3 iterations), and subsequently filtered with a 3D isotropic gaussian kernel of 4 mm FWHM, reconstructing 127 contiguous 344 × 344 axial slices, with 1.0 × 1.0 × 2.0 mm voxel size and a final spatial resolution of ~5.8 mm along the XYZ axes.

Fat and water maps derived from a dual-echo Dixon-based sequence (TR/TE1-TE2 3.3/1.23-2.46 ms) were used for MR-based attenuation^47^.

Simultaneously to PET data acquisition, a T2*-weighted single-shot EPI sequence (TR/TE = 2040/30 ms; 37 axial slices with 0.5 mm gap; flip angle = 90°; voxel-size 3.5 × 3.5 × 3 mm^3^; 600 time points; acquisition matrix 64 × 64) was run, which was used both for prospectic motion correction of PET data and to measure FC.

Immediately after the PET/RS-fMRI acquisition, a Magnetization-Prepared Rapid Acquisition Gradient-Echo sequence (MPRAGE; TR/TE/TI 2300/2.3/900 ms; 176 contiguous sagittal planes; acquisition matrix 256 × 248; voxel size 1 × 1 × 1 mm^3^; flip angle 8°) was acquired to provide anatomical reference.

### ROI definition

pPDU and FC of the six main hubs of the CSTCL (pre- and post-central gyri, posterior putamen, caudate, globus pallidus, and thalamus) on the most affected side (MAS, defined as the side with the lowest pPDU), were simultaneously measured from PET/MRI data.

To this end, bilateral subject-specific GM ROIs of the putamen, caudate, globus pallidum, and thalamus were obtained segmenting each subject’s MPRAGE volume using FIRST^48^. The MPRAGE volume was then simultaneously spatially segmented and normalized to the MNI space by the unified segmentation approach available in SPM12^49^, and the resulting normalization matrix was applied to the basal ganglia and thalamus ROIs, to obtain normalized subject-specific bilateral ROIs. Putamen ROIs were then automatically edited zeroing the ROI voxels in front of the anterior commissure (i.e. voxels with MNI Y coordinates >0 mm) to obtain the right and left posterior putamen ROIs, while normalized brain tissues obtained through MPRAGE segmentation were used to define subject-specific GM, WM and CSF ROIs.

In addition, pre- and post-central cortex ROIs defined in the MNI space were derived from the Automated Anatomical Labeling (AAL) atlas^50^.

For ROI definition, FIRST and AAL were selected respectively for deep GM segmentation and cortical labelling, based on their proven accuracy, which equaled or compared favorably to other widely used tools performing the same tasks^51,52^.

### Pre-processing

PET data were preliminarily corrected for head movements based on the EPI images using the scanner’s motion correction routine^15^.

For subsequent processing, for each study the three datasets (motion-corrected ^18^F-DOPA-PET, RS-fMRI, and MPRAGE) were spatially normalized to the MNI space using SPM12^53^, using as templates respectively a publicly available ^18^F-DOPA template (average of the normalized ^18^F-DOPA PET studies form 12 healthy controls, https://www.nitrc.org/projects/spmtemplates), and the EPI and T1 templates available in SPM12.

For the subsequent analysis, right and left pPDU were preliminarily calculated for each subject by applying the posterior putamen ROIs to the normalized ^18^F-DOPA volumes, and normalized by the occipital uptake derived from a sphere with 10 mm radius centered at [0,79,0] MNI coordinates.

RS-fMRI data were processed using the Functional Connectivity toolbox (CONN, v. 20.b, McGovern Institute for Brain Research, Massachusetts Institute of Technology, http://www.nitrc.org/projects/conn). Preprocessing steps of RS-fMRI data included: the removal of the first ten time points (to allow for instability of the initial MRI signal); rigid-body co-registration of all volumes to the first one to get rid of intra-sequence movements^54^; a slice timing correction, temporal band-pass filtering (using a 0.008–0.09 Hz band-pass filter), spatial smoothing using a 6 mm FWHM Gaussian kernel; linear detrending.

Resulting pre-processed EPI volumes were then flipped as needed, so that for each subject the side of the brain with the lower pPDU (Most Affected Side - MAS) corresponded to the right.

For all pre-processing steps, visual assessment of accuracy of segmentation and normalization results was performed by an experienced operator.

### FC map generation

Subsequently, for each of the six ROIs corresponding to the main hubs of the right cortico-striato-nigro-thalamo-cortical loop and for each patient, the corresponding correlation map of the BOLD signal across the brain was generated. The calculation of each ROI FC map included in the model the main components of the time courses of White Matter (WM) and Cerebrospinal Fluid (CSF) signals as per the CompCor procedure^55^, and the six parameters (translations and rotations along the *X*, *Y*, and *Z* axes) of spatial transformation derived from the coregistration step^56^, to remove spurious correlations related to scanner drift or patients’ motion.

In addition, vectors set to 1 for the outlier scans (i.e. volumes with >0.9 mm of relative movement, or >5 *z* values in mean signal change over the whole brain voxels) and the preceding and two following ones, were included in the model to further remove motion effects, as per the ‘scrubbing’ procedure^56^.

### Statistical analysis

Comparison of pPDU between PD and ET patients was carried out by Student’s *t* test, and correlation of pPDU with clinical scores in the PD patients was assessed by partial correlation, removing the effect of age and sex.

For each seed, the correlation between FC and pPDU was tested voxelwise separately for the ET and PD groups within the framework of the general linear model. In the model, the average mean displacement (computed as the root-mean-square of the translation parameters at each time point) and the number of analyzed time points, were included as nuisance covariates to remove potential residual movement effects, along with age and sex.

Results corrected for Family-Wise Error (FWE) at cluster level, following cluster-forming threshold of *p* = 0.001^57^, were considered significant for *p* < 0.0083 (0.05 corrected for *n* = 6 seeds according to Bonferroni). For each seed, both contrasts (direct and inverse correlation between FC and pPDU) were tested.

Finally, for each contrast showing a significant direct or inverse correlation between FC and pPDU in any of the two patient groups at the preceding analysis, the mean correlation coefficient of the resulting clusters was extracted for each patient, and differences between the two groups in the correlation of FC with pPDU, as well as the relationship in PD patients between FC and the UPDRS-ME and H&Y (available in 35 PD patients), were assessed. In particular, differences between the two groups in the correlation between FC and pPDU were assessed for each contrast by interaction analysis within the general linear model, including in the model age, sex, mean motion, number of analyzed time points, group, pPDU, and the group-by-pPDU interaction terms.

Similarly, for each contrast correlations of the mean resulting clusters’ FC with the clinical scores were assessed by stepwise linear regression analysis, forcing in the model mean motion and number of analyzed time points (to take into account any residual effect of movements during MRI scan), and including in the model the age and sex. Where significant correlations emerged, the same analysis was carried out forcing also pPDU in the model, to test if FC added to nigrostriatal degeneration in determining the score.

For both pPDU-by-group interaction and correlation with clinical score analyses, *p* = 0.05 corrected for multiple comparisons according to Bonferroni was considered significant.

## Data Availability

The dataset for the present study is not publicly available as it contains information that could breach research participant privacy/consent, but is available from the corresponding author upon reasonable requests from qualified researchers, within the limitations of the provided informed consent. Every request will be reviewed by the Institutional Review Board of the University “Magna Graecia” of Catanzaro, Italy, and the requesting researcher will need to sign a data access agreement after approval.

## Code availability

Image analysis was carried out using a combination of freely available image processing tools. For deep GM segmentation FSL’s FIRST (https://fsl.fmrib.ox.ac.uk/fsl/fslwiki/FIRST) was used. For MRI segmentation and both PET and MRI spatial normalization SPM 12 (https://www.fil.ion.ucl.ac.uk/spm/software/spm12) was used, and resulting GM maps were labelled using the AAL atlas provided with SPM.

RS-fMRI data were processed using the Functional Connectivity toolbox (CONN, v. 20.b, McGovern Institute for Brain Research, Massachusetts Institute of Technology, http://www.nitrc.org/projects/conn). Interaction and linear regression analyses on ROI data were carried out using the corresponding functions in SPSS rel. 23.0.0.0 (https://www.ibm.com/analytics/spss-statistics-software). No in-house software was used for this work.
